# Is Routine Double-J Stenting Necessary in Pediatric Pyeloplasty?

**DOI:** 10.7759/cureus.105170

**Published:** 2026-03-13

**Authors:** Sebastian G Tobia González, Anna Bujons Tur, Tiago Rosito, Ignacio P Tobia Gonzalez, Jose Fadil Iturralde

**Affiliations:** 1 Urology, Driscoll Children’s Hospital, Corpus Christi, USA; 2 Pediatric Urology, Fundació Puigvert, Barcelona, ESP; 3 Urology, Hospital de Clínicas de Porto Alegre, Porto Alegre, BRA; 4 Urology, Universidad Nacional de La Plata, La Plata, ARG; 5 Urology, Sanatorio Parque, Rosario, ARG

**Keywords:** pediatric urology, postoperative complications, pyeloplasty, ureteral stents, ureteropelvic junction obstruction

## Abstract

Background

The role of routine internal drainage with a double-J (DJ) ureteral stent in pediatric pyeloplasty remains controversial. While stents are frequently used in minimally invasive approaches, their necessity in open surgery is debated, particularly regarding their impact on postoperative complications. This study aimed to evaluate whether the routine use of DJ stents influences postoperative complication rates in pediatric pyeloplasty within a multicenter international collaborative cohort.

Methodology

We performed a retrospective analysis of a pooled multicenter database including pediatric patients undergoing pyeloplasty (open, laparoscopic, and robotic) across four international institutions. Patients were categorized according to DJ stent use. The primary outcome was the overall postoperative complication rate. Secondary outcomes included urinary tract infection (UTI), reoperation, and length of hospital stay. Laparoscopic and robotic cases, in which DJ stents were routinely used, served as contemporary reference controls. Comparative analyses were performed between stented and non-stented cases, with statistical significance defined as a p-value <0.05.

Results

A total of 231 pediatric pyeloplasty cases with complete documentation regarding DJ stent utilization (presence or absence) were included in the comparative analysis. Open pyeloplasty cases were performed with or without DJ stenting, while minimally invasive approaches predominantly included routine stenting. Complication rates were 26.7% in the stented group and 20.4% in the non-stented group (p = 0.39). Reoperation rates were 3.7% and 4.5%, respectively (p = 0.81). No statistically significant differences were observed in rates of UTI, reoperation, or length of stay. Outcomes remained consistent across surgical approaches and stent utilization strategies.

Conclusions

Routine use of a DJ stent does not reduce postoperative complications in pediatric pyeloplasty. In this multicenter cohort, comparable outcomes were observed with and without internal drainage. These findings suggest that routine DJ placement is likely unnecessary in most open pyeloplasty cases and support a selective rather than universal stenting strategy.

## Introduction

Ureteropelvic junction obstruction (UPJO) is one of the most common causes of hydronephrosis requiring surgical intervention in pediatric patients. Dismembered pyeloplasty remains the gold standard treatment, traditionally performed via an open approach and, more recently, through minimally invasive laparoscopic and robotic techniques. Across all approaches, reported success rates exceed 90%, with low rates of recurrent obstruction and acceptable morbidity [1,2].

Despite the well-established surgical principles of pyeloplasty, the optimal strategy for postoperative urinary drainage remains controversial [3,4]. Internal drainage using a double-J (DJ) ureteral stent is widely practiced, particularly in minimally invasive surgery, where routine stenting has become standard in many centers. However, the necessity of routine stenting in open pyeloplasty continues to be debated. While proponents argue that DJ stents may reduce urinary leakage and transient obstruction, critics highlight the need for secondary anesthesia for removal, potential stent-related discomfort, infection risk, and additional healthcare utilization [5].

Existing literature has reported comparable overall success and complication rates between stented and non-stented pyeloplasty, although heterogeneity in surgical approach, institutional protocols, and surgeon preference limits definitive conclusions [3,4,6]. Furthermore, many published series represent single-center experiences, and few analyses incorporate contemporary minimally invasive techniques as contextual comparators [1,7].

Given the variability in international practice patterns and the lack of consensus regarding routine internal drainage, we aimed to evaluate the impact of DJ stent use on postoperative outcomes in pediatric pyeloplasty using a pooled multicenter international database. We hypothesized that routine DJ stenting does not significantly reduce overall postoperative complication rates, particularly in open pyeloplasty, and that outcomes are comparable across surgical approaches.

## Materials and methods

A retrospective, multicenter, cohort study was conducted using a pooled international database of pediatric patients who underwent pyeloplasty for UPJO. The dataset included consecutive cases collected between 2000 and 2024 from four tertiary pediatric urology centers participating in an international collaborative registry. All patient data were fully anonymized before analysis, and no center identifiers were retained in the analytical dataset to ensure institutional confidentiality.

Patients younger than 18 years of age who underwent primary dismembered pyeloplasty with clearly documented intraoperative use or non-use of a DJ ureteral stent were eligible for inclusion. Cases were excluded if they involved redo pyeloplasty, lacked documentation regarding stent utilization, or used alternative urinary diversion strategies not comparable to internal DJ stenting.

Surgical approaches included open, laparoscopic, and robotic-assisted pyeloplasty. Open procedures were performed with selective use of DJ stents according to surgeon preference, whereas minimally invasive approaches were predominantly performed with routine internal DJ stenting and served as contemporary reference controls.

Patients were categorized into the following two groups based on intraoperative drainage strategy: those receiving a DJ stent (stented group) and those undergoing pyeloplasty without internal stenting (non-stented group). The primary comparison focused on open pyeloplasty performed with versus without DJ stenting, while minimally invasive cases were analyzed to contextualize modern practice patterns.

The primary outcome was the overall postoperative complication rate. Secondary outcomes included urinary tract infections (UTIs), reoperation, length of hospital stay, and operative time. Operative time was defined as the interval from skin incision to completion of skin closure, as documented in the operative report. Postoperative complications were defined as any deviation from the expected postoperative course requiring medical or surgical intervention.

Continuous variables were reported as mean ± standard deviation or median with interquartile range, depending on distribution. Categorical variables were expressed as n (%). Group comparisons were performed using the chi-square or Fisher’s exact test for categorical variables and Student’s t-test or Mann-Whitney U test for continuous variables, as appropriate. Odds ratios (ORs) with 95% confidence intervals (CIs) were calculated for key outcomes. A p-value <0.05 was considered statistically significant. Statistical analyses were performed using SPSS version 26 (IBM Corp., Armonk, NY, USA).

The study was approved by the institutional research ethics committee of a participating tertiary pediatric center and complied with the Declaration of Helsinki and CIOMS guidelines. Given the retrospective and de-identified nature of the dataset, the requirement for informed consent was waived.

## Results

A total of 231 pediatric pyeloplasty cases with complete documentation of DJ stent utilization were included in the comparative analysis. Of these, 187 (81.0%) patients underwent pyeloplasty with DJ stent placement, and 44 (19.0%) underwent surgery without internal stenting.

Baseline demographic characteristics and surgical approach distribution are presented in Table 1. Patients in the non-stented group were significantly younger than those in the stented group (median age 6.0 vs. 10.0 years, p < 0.001). Sex distribution was similar between groups, with females comprising 54 (28.9%) in the stented group and 14 (31.8%) in the non-stented group (p = 0.84). The prevalence of prenatal hydronephrosis did not differ significantly between groups (p = 1.00). As expected, the surgical approach was strongly associated with stent utilization (p < 0.001), with laparoscopic and robotic procedures predominantly performed with routine DJ stenting, while the majority of non-stented cases were open procedures.

**Table 1:** Baseline demographics and surgical approach by stent utilization. Values are presented as n (%) unless otherwise specified. ^†^: Age had missing values: available in 198/231 overall (DJ: 154; no DJ: 44). Mann–Whitney U test (U) and Welch t-test (t) are shown for transparency; report one consistently in text (recommend Mann–Whitney). ^‡^: Prenatal hydronephrosis had missing values: available in 179/231 overall (DJ: 139; no DJ: 40). The chi-square test was used among cases with available data. IQR = interquartile range; DJ = double-J

Variable	DJ (n = 187)	No DJ (n = 44)	Statistic	P-value
Age, years, median (IQR)^†^	10.0 (6.0–12.8) (n = 154)	6.0 (2.0–9.0) (n = 44)	U = 4881.5	<0.001
Sex			χ² = 0.04	0.84
Female	54 (28.9%)	14 (31.8%)		
Male	133 (71.1%)	30 (68.2%)		
Prenatal hydronephrosis^‡^			χ² = 0.00	1.00
Yes	30 (21.6%)	8 (20.0%)		
No	109 (78.4%)	32 (80.0%)		
Surgical approach (Tipo Cx)			χ² = 66.03	<0.001
Open (1)	47 (25.1%)	40 (90.9%)		
Laparoscopic (2)	89 (47.6%)	4 (9.1%)		
Robotic (3)	51 (27.3%)	0 (0.0%)		

Comparative postoperative outcomes are summarized in Table 2. Overall, postoperative complications occurred in 59 of 231 (25.5%) patients across the cohort. Complications were observed in 50 (26.7%) patients in the stented group and 9 (20.4%) in the non-stented group. This difference was not statistically significant (chi-square = 0.72, p = 0.39). The OR for postoperative complications associated with DJ stenting was 1.42 (95% CI = 0.64-3.16), indicating no protective effect of routine internal drainage. Operative time differed between groups. Median operative time was 140 minutes (IQR = 108-180) in the DJ stented group and 108 minutes (IQR = 94-120) in the non-stented group. Median length of hospital stay was three days (IQR = 2-5) in the stented group and 5 days (IQR = 5-5) in the non-stented group.

**Table 2:** Postoperative outcomes according to DJ stent utilization. Values are presented as n (%) unless otherwise indicated. Continuous variables are reported as median (IQR). IQR = interquartile range; DJ = double-J; OR = odds ratio; CI = confidence interval

Outcome	DJ stent (n = 187)	No stent (n = 44)
Overall complications	50 (26.7%)	9 (20.4%)
Reoperation	7 (3.7%)	2 (4.5%)
Urinary tract infection	13 (7.0%)	3 (6.8%)
Operative time (minutes), median (IQR)	140 (108–180)	108 (94–120)
Length of stay (days), median (IQR)	3 (2–5)	5 (5–5)

Reoperation rates were low in both groups. Reintervention was required in seven (3.7%) patients in the stented group and two (4.5%) in the non-stented group, with no statistically significant difference (chi-square = 0.06, p = 0.81). The corresponding OR was 0.82 (95% CI = 0.16-4.08).

No statistically significant differences were identified between groups in rates of UTI, length of hospital stay, or operative time. Overall, the use of a DJ stent was not associated with improved postoperative outcomes, and complication rates remained comparable regardless of stent utilization or surgical approach.

## Discussion

The optimal strategy for postoperative urinary drainage in pediatric pyeloplasty remains an area of ongoing debate. Although internal DJ ureteral stents are widely used, particularly in minimally invasive approaches, their routine necessity in open pyeloplasty has not been definitively established [3,4]. In the present series, postoperative complications occurred in 50 (26.7%) stented cases and 9 (20.4%) non-stented cases (p = 0.39), with similarly low reoperation rates of 7 (3.7%) and 2 (4.5%) (p = 0.81). These findings suggest that routine DJ stenting does not confer a statistically significant reduction in postoperative morbidity.

The primary comparison of interest, open pyeloplasty performed with versus without DJ stenting, demonstrated comparable outcomes between groups. These findings suggest that routine DJ stenting in open pyeloplasty is likely unnecessary in most cases, as it was not associated with a statistically significant reduction in postoperative complications or reoperation rates [7]. Importantly, the absence of even a trend toward statistical benefit further supports reconsideration of routine stenting in open surgery. Complication profiles were similar, and most events were minor and managed conservatively. Rates of reoperation were low across both groups, further supporting the safety of selective stent omission in appropriately selected cases.

Minimally invasive approaches in this cohort predominantly incorporated routine DJ stenting, reflecting contemporary surgical practice. Despite systematic stent placement in laparoscopic and robotic procedures, overall complication rates were not lower compared to open cases performed without routine stenting [3,4]. This observation reinforces the notion that internal drainage alone does not appear to be the determining factor in postoperative morbidity.

Several theoretical benefits of DJ stents have been proposed, including reduced urinary leakage, prevention of transient postoperative obstruction, and protection of the anastomosis [5,8]. Conversely, stent-related drawbacks include the need for secondary anesthesia for removal, patient discomfort, irritative urinary symptoms, risk of infection, and increased healthcare utilization [9]. The absence of a demonstrable reduction in global complication rates in the present study suggests that these potential benefits may not translate into meaningful clinical superiority when applied routinely.

Our findings align with previously published series reporting comparable success rates between stented and non-stented pediatric pyeloplasty [1,3,4]. However, many prior studies were single-center experiences or focused on a single surgical approach [6,7]. The strength of the present study lies in its multicenter international design and inclusion of open, laparoscopic, and robotic techniques, allowing broader contextual interpretation of practice patterns.

The concept of selective rather than routine stenting is supported by these data. In open pyeloplasty, where direct visualization and meticulous anastomotic technique are achievable, omission of routine internal drainage may be reasonable without compromising patient safety. Individualized decision-making based on intraoperative findings, such as anastomotic tension, tissue quality, or significant inflammation, may represent a more rational approach than universal stent placement.

Limitations

This study has limitations inherent to its retrospective design. Although data were pooled from multiple centers, the absence of center-level identifiers prevented stratified institutional analysis. The decision to place a DJ stent in open cases was surgeon-dependent and may have introduced selection bias, as patients perceived to be at higher risk could have preferentially received internal drainage. In addition, age differed significantly between groups, and stent utilization was strongly associated with surgical approach. Because multivariable adjustment was not performed, these factors may confound the observed comparisons between stented and non-stented groups. Consequently, the results should be interpreted as observational associations rather than evidence of a causal effect of stent placement.

Additionally, complication grading relied on clinical documentation within the database, which may limit the granularity of severity assessment. The imbalance in group size between stented and non-stented patients may have limited the statistical power to detect small differences. In addition, stent utilization was closely associated with surgical approach, as minimally invasive procedures were almost uniformly performed with routine stenting. Consequently, comparisons between stented and non-stented groups may be partially confounded by surgical approach and should be interpreted with caution. Despite these limitations, the large pooled cohort and consistency of findings across surgical approaches strengthen the validity of the conclusions.

## Conclusions

In this multicenter international cohort, routine DJ stenting was not associated with a reduction in postoperative complications, UTI, or reoperation following pediatric pyeloplasty. Comparable outcomes were observed across surgical approaches and regardless of stent utilization. These findings suggest that comparable outcomes may be achieved with selective rather than routine DJ placement in open pyeloplasty, although the results should be interpreted in light of potential selection bias and confounding. Adoption of a selective stenting strategy may help reduce additional procedures and resource utilization without compromising patient safety.
